# Structures of pyruvate kinases display evolutionarily divergent allosteric strategies

**DOI:** 10.1098/rsos.140120

**Published:** 2014-09-24

**Authors:** Hugh P. Morgan, Wenhe Zhong, Iain W. McNae, Paul A. M. Michels, Linda A. Fothergill-Gilmore, Malcolm D. Walkinshaw

**Affiliations:** Centre for Translational and Chemical Biology, School of Biological Sciences, University of Edinburgh, Michael Swann Building, The King's Buildings, Mayfield Road, Edinburgh EH9 3JR, UK

**Keywords:** apoenzyme, effector bound enzyme, *Leishmania*, specificity constant, tetramer stability, *Trypanosoma*

## Abstract

The transition between the inactive T-state (apoenzyme) and active R-state (effector bound enzyme) of *Trypanosoma cruzi* pyruvate kinase (PYK) is accompanied by a symmetrical 8° rigid body rocking motion of the A- and C-domain cores in each of the four subunits, coupled with the formation of additional salt bridges across two of the four subunit interfaces. These salt bridges provide increased tetramer stability correlated with an enhanced specificity constant (*k*_cat_/*S*_0.5_). A detailed kinetic and structural comparison between the potential drug target PYKs from the pathogenic protists *T. cruzi*, *T. brucei* and *Leishmania mexicana* shows that their allosteric mechanism is conserved. By contrast, a structural comparison of trypanosomatid PYKs with the evolutionarily divergent PYKs of humans and of bacteria shows that they have adopted different allosteric strategies. The underlying principle in each case is to maximize (*k*_cat_/*S*_0.5_) by stabilizing and rigidifying the tetramer in an active R-state conformation. However, bacterial and mammalian PYKs have evolved alternative ways of locking the tetramers together. In contrast to the divergent allosteric mechanisms, the PYK active sites are highly conserved across species. Selective disruption of the varied allosteric mechanisms may therefore provide a useful approach for the design of species-specific inhibitors.

## 2. Background

**Figure 1.**
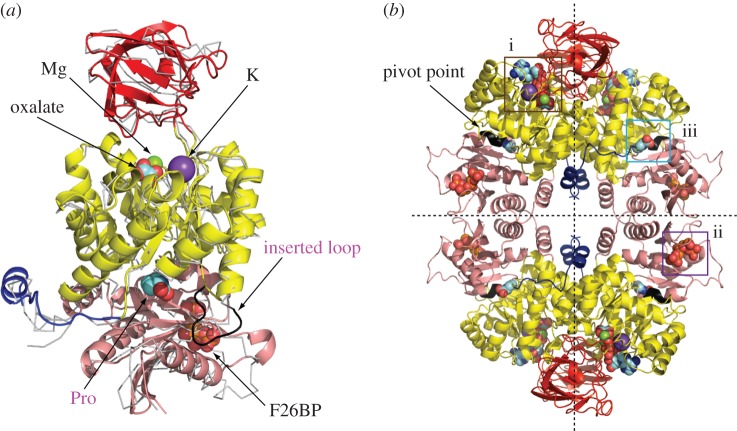
Structure of PYK and identification of regulatory binding sites. (*a*) A monomer of the *Tc*PYK-F26BP-OX-Mg complex (shown as cartoon, PDB ID: 4ks0) superposed onto the human M1PYK-Pro structure (shown as ribbon, PDB ID: 3n25 [1]). The *Tc*PYK monomer is coloured to highlight domains; N (blue, residues 1–18), A (yellow, residues 19–89 and 188–358), B (red, residues 90–187) and C (pink, residues 359–499). Space-filling representations are used to show oxalate, F26BP and proline (Pro), as well as Mg and K. The inserted loop (Ser^100^-Asp-Pro-Ile-Leu-Tyr^105^) from human M1PYK is shown in black and is conserved in human M2PYK. (*b*) A representative PYK tetramer (*Tc*PYK PDB ID: 4ks0 is shown here) highlighting the (i) active, (ii) effector and (iii) amino acid binding sites. The active site has bound oxalate, ATP, Mg and K; the effector site bound F26BP; and the attenuating site bound proline. The pivot region about which the protomers rotate is shown in black. The large (A–A) and small (C–C) interfaces between monomers are shown as vertical and horizontal dashed lines, respectively.

**Figure 2.**
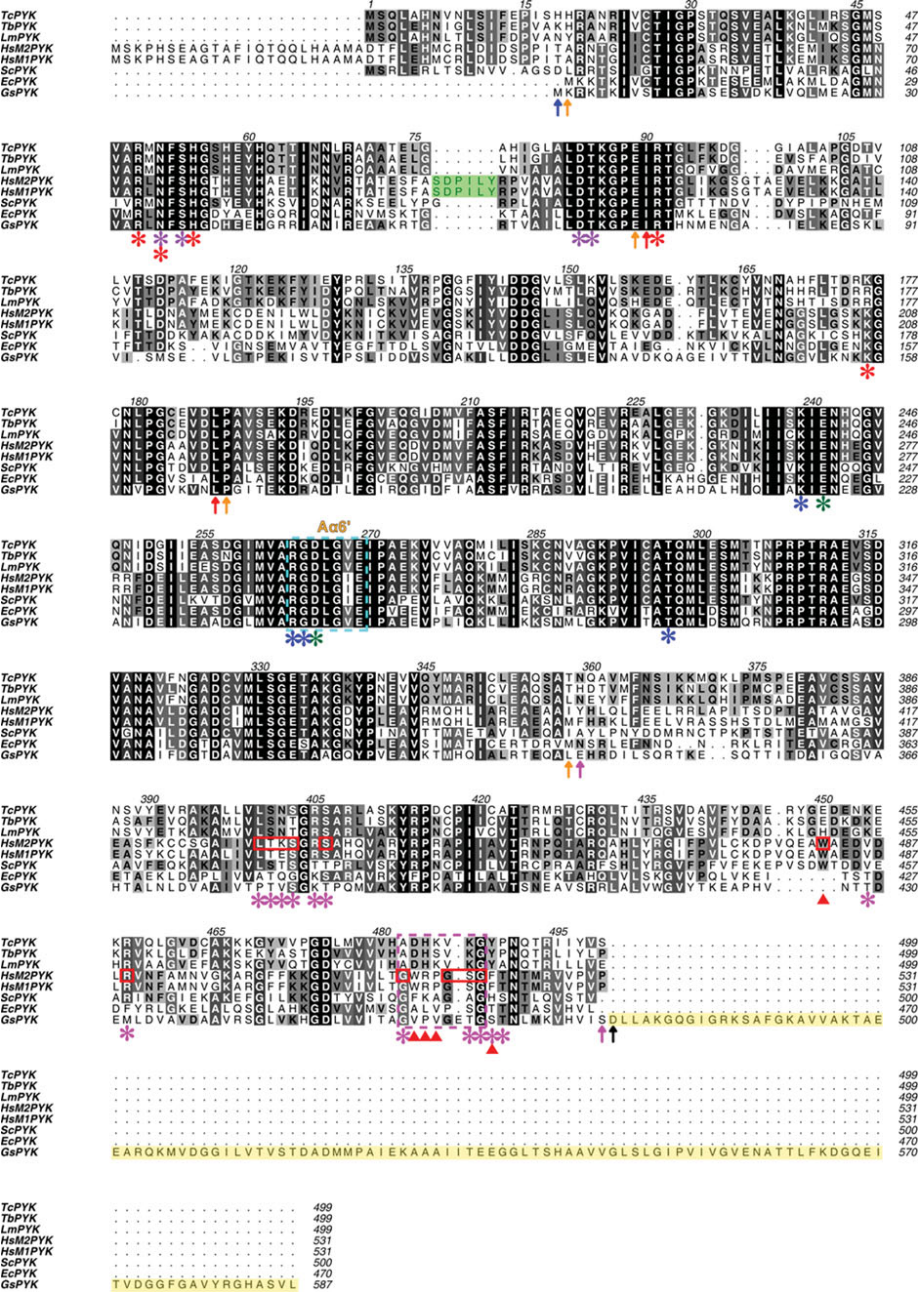
Sequence alignment of pyruvate kinases from trypanosomatids, humans, yeast and bacteria. The sequence alignment was performed using the program Clustal Omega at the European Bioinformatics Institute [3,4]. Domain boundaries are indicated by vertical arrows in domain-specific colours: N-terminal domain (blue), A-domain (yellow), B-domain (red) and C-domain (pink). The extra C-terminal domain (C'-domain) which potentially plays a role in tetramer stabilization is shaded in yellow. The conservation of the residues is indicated by shading from black (identical in eight sequences) to grey (conserved in five, six or seven) to white (low or no conservation). Residue numbers corresponding to each PYK are listed after the sequences. Residue numbers corresponding to trypanosomatid PYKs are also listed above the sequences. The methionine residues at the N-termini correspond to the start codons and do not necessarily occur in the mature proteins. In trypanosomatid PYKs, the amino acids involved in divalent metal binding (oxalate-coordinating metal, Mg-1 site) (green asterisk), potassium metal binding (purple asterisk), oxalate binding (blue asterisk), nucleotide binding (red asterisk) and effector F26BP binding (pink asterisk) are indicated by asterisks. Residues 263–269 of the small *α*-helix A*α*6′ which are involved in allosteric regulation and in binding divalent metal and oxalate are indicated by a dashed box (cyan). The effector loop residues are indicated by a pink dashed box. The F16BP binding residues in human M2PYK are shown by red boxes. The residues involved in stacking interactions to stabilize the effector loop of human M2PYK are indicated by triangles in red. The inserted loop (Ser^100^-Asp-Pro-Ile-Leu-Tyr^105^) from human M2PYK/M1PYK is shaded in green. The figure was generated using the program Aline [5]. *Tc*, *Trypanosoma cruzi* (UniProtKB: Q4D9Z4); *Tb*, *Trypanosoma brucei* (UniProtKB: P30615); *Lm*, *Leishmania mexicana* (UniProtKB: Q27686); *Hs*, *Homo sapiens* (UniProtKB: P14618); *Sc*, *Saccharomyces cerevisiae* (baker's yeast) (UniProtKB: P00549); *Ec*, *Escherichia coli* (UniProtKB: P0AD61); *Gs*, *Geobacillus stearothermophilus* (UniProtKB: Q02499).

Pyruvate kinase (PYK, EC 2.7.1.40) catalyses the final step in glycolysis, namely the transfer of a phospho group from phospho*enol*pyruvate (PEP) to ADP to form pyruvate and ATP. PYK exists primarily as a homotetramer with subunits of 50–60 kDa (depending on species), each of which is composed of four domains: the N-terminal (not present in bacteria), A-, B- and C-domains (figure 1*a*). An additional C'-domain is also found in PYKs from *Geobacillus stearothermophilis* [2] (figure 2) and *Staphylococcus aureus* [6]. The active site is nestled between the A- and B-domains and is located approximately 39 Å from the effector site which is located in the C-domain. In the tetramer, adjacent C-domains form the C–C or ‘small’ interface, and neighbouring A-domains form the A–A or ‘large’ interface. The B-domain contributes a mobile lid at one end of the (*α*/*β*)_8_-barrelled A-domain and modulates access to the active site.

The overall tetrameric structure of PYK is highly conserved across distant phylogenetic groups; however, strategies for the regulation of PYK activity vary substantially between species [2,7–11]. Three distinct ligand-binding sites with affinities for a range of small molecules have been identified in PYK tetramers and are particularly well characterized in mammals, trypanosomatids, yeast and bacteria (figure 1*b*). These sites are designated here as (i) the active site, (ii) the effector site, and (iii) the amino acid binding site. Most PYKs display cooperative binding of the substrate PEP at the active site, with characteristic sigmoid binding curves and Hill coefficients>1 (e.g. table 1). In this way, glycolytic flux can be augmented in response to the presence of elevated levels of PEP.

**Table 1.** Comparison of kinetic properties of PYKs. *k*_cat_ values in s^−1^; *k*_cat_/*S*_0.5_ values in *s*^−1^ *mM*^−1^; *h*, Hill coefficient; n.d., not done. *Tc*, *T. cruzi*; *Tb*, *T. brucei*; *Lm*, *L. mexicana*; *Hs*, *Homo sapiens*; *Sc*, *Saccharomyces cerevisiae*; *Ec*, *Escherichia coli*; *Gs*, *Geobacillus stearothermophilus.*

ligand	modulator	kinetic properties parameter	*Tc*PYK 25°C	*Tb*PYK 25°C	*Lm*PYK 25°C	*Hs*RPYK 37°C	*Hs*LPYK 25°C	*Hs*M2PYK 37°C	*Hs*M1PYK 37°C	*Sc*PYK 25°C	*Ec*PYK 25°C	*Gs*PYK^*d*^
reference			this work	[12]	[13]	[14]	[15]	[16]	[16]	[17]	[18]	[2,19]
PEP	none	*S*_0.5_ (mM)	1.23±0.06	1.03	1.06	1.1	0.96	0.86	0.05	2.76	3.63	0.96
		*h*	2.06±0.16	1.88	1.5	1.6	2.0	1.2	1.0	2.93	3.2	2.2
		*k*_cat_	169±5	145	296	355	494	117	347	188	123	187
		*k*_cat_/*S*_0.5_	137	141	279	323	515	136	6939	68	40	85
	F26BP	*S*_0.5_ (mM)	0.14±0.02	0.12	0.11	n.d.	n.d.	n.d.	n.d.	n.d.	n.d.	n.d.
		*h*	1.13±0.13	1.2	1.0	n.d.	n.d.	n.d.	n.d.	n.d.	n.d.	n.d.
		*k*_cat_	212±7	231	300	n.d.	n.d.	n.d.	n.d.	n.d.	n.d.	n.d.
		*k*_cat_/*S*_0.5_	1514	1925	2727	n.d.	n.d.	n.d.	n.d.	n.d.	n.d.	n.d.
	F16BP	*S*_0.5_ (mM)	0.35±0.01	0.26	0.84^*b*^	0.18	0.3	0.10	0.05	0.22	0.08	0.28 (AMP)
		*h*	2.17±0.12	1.31	2.68^*b*^	1.05	1.6	1.0	1.0	1.7	1.0	1.0 (AMP)
		*k*_cat_	226±3	186	417^*b*^	355	494	222	357	195	160	187 (AMP)
		*k*_cat_/*S*_0.5_	646	715	496^*b*^	1972	1647	2218	7140	886	2000	668 (AMP)
ADP	none	*K*_*m*_ (mM)	0.40±0.04	0.14	0.26^*b*^	0.17	0.4	0.34	0.37^*c*^	0.25	0.30	n.d.
F26BP	none	*Ka*_0.5_ (μM)	0.030±0.001	0.01^*a*^	0.29^*b*^	n.d.	n.d.	n.d.	n.d.	n.d.	n.d.	n.d.
F16BP	none	*Ka*_0.5_ (mM)	1.24±0.24	0.05	0.52^*b*^	0.0004	0.0001	0.006	n.d.	n.d.	n.d.	n.d.
±effector		relative *k*_cat_/S_0.5_	11.1	13.7	9.8	6.1	3.2	16.3	1.0	13.0	50.0	7.9

^*a*^Value is from [20].

^*b*^Values are from [21].

^*c*^Value is from [22].

^*d*^*V*_max_ (average) is 200 μmol^−1^ min^−1^ mg^−1^[2] and converted to *k*_cat_ (187 s^−1^) assuming a monomer with mass of 56 000 g mol^−1^; all other values are obtained from [19]. The modulator for *Gs*PYK is AMP instead of F16BP. The temperature for the assay is not stated in the references.

Modulation of PYK activity by activators and inhibitors which bind to the effector site is well characterized (table 1). Fructose 1,6-bisphosphate (F16BP) is the allosteric activator of three of the four mammalian isoenzymes: RPYK (erythrocyte), LPYK (liver) and M2PYK (embryonic or tumour). The fourth isoenzyme is the constitutively active M1PYK found in skeletal muscle. The activity of M1PYK can also be attenuated by the binding of amino acids such as proline or phenylalanine at the amino acid binding site [9,23]. PYK from *Escherichia coli* is also activated by F16BP, but PYK from *Geobacillus stearothermophilus* is allosterically activated by AMP or ribose 5-phosphate (R5P) [8] which probably bind at a position corresponding to the effector site [2]. PYK from baker's yeast *Saccharomyces cerevisiae* is also activated by F16BP [17] (table 1). Interestingly, PYKs from plants and archaea appear to be unresponsive to effectors such as F16BP or R5P [24,25]. There are currently no structural data for plant or archaeal PYKs.

The ‘TriTryp’ group of trypanosomatid parasites are responsible for diseases, including sleeping sickness (caused by *Trypanosoma brucei*), leishmaniasis (caused by various species of *Leishmania*) and Chagas' disease (caused by *Trypanosoma cruzi*), and *T. brucei* PYK is a validated drug target [26]. Trypanosomatid PYKs are allosterically activated by micromolar concentrations of fructose 2,6-bisphosphate (F26BP), instead of F16BP which is effective only at millimolar concentrations [27] (table 1). The X-ray structures of PYK from *L. mexicana* [10] and *T. brucei* [12] have been determined. Here, the first apoenzyme and R-state structures for *T. cruzi* PYK (*Tc*PYK) are presented, thereby providing a detailed structural and enzymatic comparison for each of the ‘TriTryp’ family members. Analysis of the R- and T-state PYK structures from trypanosomatids, mammals and bacteria also provides an explanation of the evolutionary divergence of allosteric regulation.

## 3. Material and methods

### 3.1 Materials

ADP, PEP, oxalate (OX), F16BP, F26BP, lactate dehydrogenase (LDH; rabbit muscle), polyethylene glycol (PEG) 8000, antibiotics and buffers were obtained from Sigma-Aldrich. NADH and EDTA-free protease inhibitor mixture tablets were from Roche, glycerol was from BDH Prolabo, IPTG was from Melford and salts were from Fisher Scientific. Restriction enzymes, vector and *E. coli* competent cells were from Novagen.

N-terminal His_6_-tagged *Tc*PYK was prepared as described previously [28]. Briefly, the expression of His_6_-tagged *Tc*PYK was achieved by the T7lac promoter-driven system in *E. coli* BL21 (DE3) cells after adding IPTG to a final concentration of 1 mM. Pure protein was obtained by immobilized metal ion affinity chromatography (IMAC) followed by gel-filtration chromatography. N-terminally His_6_-tagged human pyruvate kinases (M1PYK and M2PYK) were expressed in *E. coli* BL21(DE3) cells and purified using IMAC and gel-filtration chromatography as described previously [16].

### 3.2 Enzyme activity assay and kinetics study

The activity and kinetics of *Tc*PYK were determined as described for *T. brucei* PYK (*Tb*PYK) previously [12]. Briefly, the activity of *Tc*PYK was measured by following the decrease in NADH absorbance at 339 nm, where one activity unit is defined as the conversion of 1 μmol substrate per minute under standard conditions. The assay was performed at 298 K in 100 μl reaction mixtures containing 50 mM triethanolamine (TEA) buffer, pH 7.2, 50 mM KCl, 10 mM MgCl_2_, 10% glycerol, 0.5 mM NADH and 5 μg (3.2 U) of LDH. The turnover number (*k*_cat_) of *Tc*PYK was calculated by the enzyme specific activity divided by the subunit molar mass of 56769.1 (g mol^−1^). Enzyme kinetics with regard to ADP was studied at saturating concentrations of PEP (10 mM) and variable concentrations of ADP (from 0 to 2.5 mM). Enzyme kinetics with regard to PEP was studied at saturating concentrations of ADP (2.5 mM) and variable concentrations of PEP (from 0 to 10 mM) in the presence or the absence of 1 μM F26BP, or in the presence or the absence of 4.5 mM F16BP. Enzyme kinetics with regard to the effector F26BP was studied at 2.5 mM ADP, 0.4 mM PEP and variable concentrations of F26BP (from 0 to 1 μM), while enzyme kinetics with regard to the effector F16BP was studied at 2.5 mM ADP, 0.7 mM PEP and variable concentrations of F16BP (from 0 to 4.5 mM). The Michaelis–Menten equation or its extended equations were fitted to the experimental data to calculate the values of the kinetic parameters. The parameter *S*_0.5_ is used instead of *K*_*m*_ when the substrate binding shows cooperativity. The parameter *Ka*_0.5_ refers to the concentration of activator at which half the maximal activation is observed.

### 3.3 Thermal shift assay (differential scanning fluorimetry)

Melting temperature (*T*_m_) was determined by a thermal shift assay using a fluorescence method as described for *Tb*PYK previously [12]. For *Tc*PYK and *Tb*PYK, the assay buffer contained 50 mM TEA, pH 7.2, 100 mM KCl, 10 mM MgCl_2_, 10× SYPRO Orange dye (the dye was supplied by Invitrogen (catalogue number S6650) at 5000× concentration in DMSO, and diluted in assay buffer for use), 4 μM *Tc*PYK or *Tb*PYK, in the presence or the absence of 1 μM F26BP.

### 3.4 Dynamic light scattering

The *T*_*m*_ values for human M2PYK and human M1PYK were also determined by monitoring changes in light scattering with increasing temperature. Five microlitres of human M2PYK or human M1PYK (10 mg ml^−1^), 5 μl of 10× metals buffer (500 mM TEA, pH 7.2, 500 mM MgCl_2_, 1 M KCl) and 1 μl of each ligand (50 mM stocks prepared in 100 mM TEA buffer, pH 7.2), the final volume was then adjusted to 50 μl using dilution buffer (10 mM TEA (pH 7.2) and 2% glycerol).

A Zetasizer Auto Plate Sampler was used to determine the Z-average molecular ‘size’ in terms of the hydrodynamic diameter in solution. Changes in the Z-average or particle diameter with increased temperature (293–353 K in increments of 1 K) were monitored in automated mode (typically requiring a measurement duration of 150 s; 13 acquisitions were determined for each run and repeated in triplicate). The resulting data were then analysed using the manufacturer's software provided (Malvern Instruments Ltd, Malvern, UK).

### 3.5 Crystallization, data collection and structure determination

Purified *Tc*PYK aliquots (30 mg ml^−1^) were diluted to 15 mg ml^−1^ using a buffer containing 50 mM TEA (pH 7.2) or supplemented with 3.5 mM ATP, 3.5 μM F26BP and 3.5 mM oxalate. Single crystals of *Tc*PYK or *Tc*PYK complexed with F26BP, Mg^2+^ and oxalate (there was no evidence of ATP from the crystal structure) were obtained at 277 K by vapour diffusion using the hanging drop technique. The drops were formed by mixing 1.5 μl of protein solution with 1.5 μl of a well solution composed of 8–18% PEG 8000, 20 mM TEA buffer (pH 7.2), 50 mM MgCl_2_, 100 mM KCl and 10–20% glycerol. The drops were equilibrated against a reservoir filled with 0.5 ml of well solution.

Prior to data collection, crystals were equilibrated for 14 h over a well solution composed of 8–18% PEG 8000 (2% above the crystallization conditions), 20 mM TEA buffer (pH 7.2), 50 mM MgCl_2_, 100 mM KCl and 25% glycerol, which eliminated the appearance of ice rings. Intensity data were collected at the Diamond synchrotron radiation facility in Oxfordshire, UK on beamline I04 (*Tc*PYK) or I03 (*Tc*PYK-F26BP-OX-Mg) to a resolution of 2.50 Å (*Tc*PYK), or 2.80 Å(*Tc*PYK-F26BP-OX-Mg). All datasets were obtained from a single crystal flash frozen in liquid nitrogen at 100 K. The *Tc*PYK structures were solved as described previously [28].

Superpositions of PYK structures were performed using both PyMOL [29] and CCP4 superpose [30,31]. CCP4 superpose was also used to calculate the allosteric rigid body rotations of *Tc*PYK from the superposition of T- and R-state tetramers (AC cores) as described previously [10]. Both RMS difference numbers and rotation matrices were calculated in the superposition process.

## 4. Results and discussion

### 4.1 *Tc*PYK and *Tb*PYK have similar kinetic parameters

Values of *Tc*PYK kinetic parameters were determined for PEP in the presence and absence of both the trypanosomatid PYK allosteric activator F26BP and the more general activator F16BP at 298 K and are summarized in table 1, together with the parameters previously reported for *Tb*PYK [12]. In the absence of F26BP, the *S*_0.5_ values for PEP were 1.23 mM and 1.03 mM for *Tc*PYK and *Tb*PYK, respectively, both exhibiting positive cooperativity (*h*>1) and similar specificity constants (*k*_cat_/*S*_0.5_ values of 137 and 141 s^−1^ mM^−1^, respectively). In the presence of F26BP, the *S*_0.5_ value for PEP decreased ninefold and the specificity constant increased 11-fold (average *k*_cat_/*S*_0.5_ value of 1514 mM^−1^ s^−1^) for both *Tc*PYK and *Tb*PYK, with positive cooperativity being completely abolished (*n*=∼1). By contrast, addition of the general effector F16BP decreased the *S*_0.5_ value for PEP fourfold and increased the specificity constant fivefold, although these effects were observed only at concentrations over 1000 times higher than that of F26BP. There are no data available for the intracellular concentrations of F16BP and F26BP in *T. cruzi*; however, *in vivo* concentrations for F16BP and F26BP have been determined for the bloodstream form of *T. brucei* as approximately 600 μM for F16BP and approximately 10 μM for F26BP (calculated from data in [32] and assuming 175 mg total cellular protein per millilitre total cellular volume [33]). In *T. cruzi*, there is an interestingly large difference in the *Ka*_0.5_ values for F26BP (0.03 μM) and F16BP (1239 μM) (table 1). The corresponding *Ka*_0.5_ values for *T. brucei* are 0.01 and 50 μM. Based on the suggested relative cellular concentrations of the FBPs and their PYK affinities, it is not out of the question that F16BP may also contribute to PYK activation in trypanosomatids.

**Figure 3.**
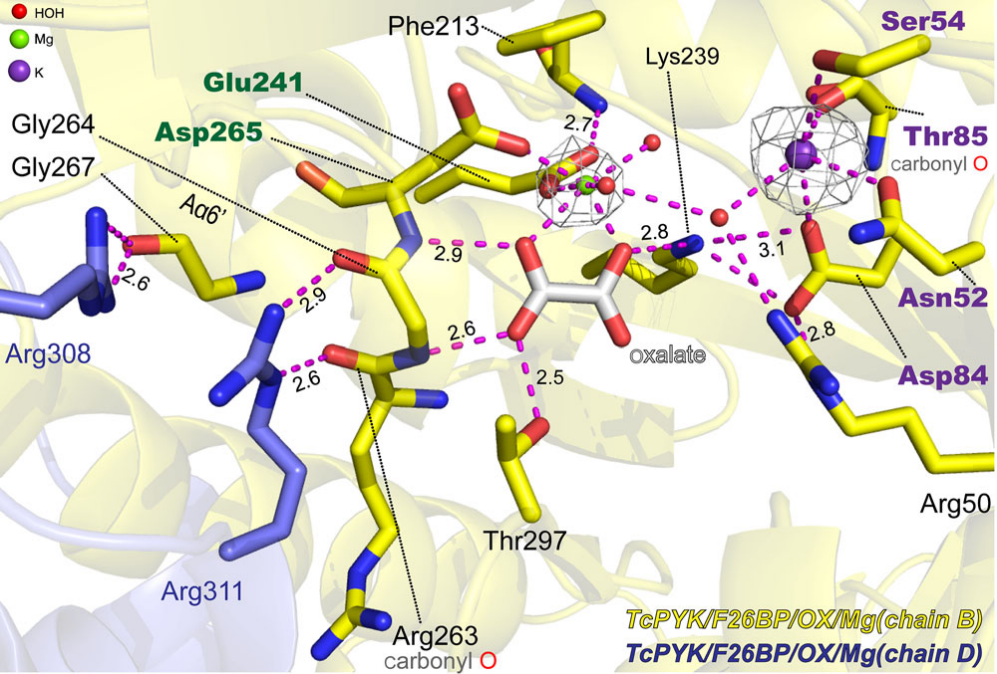
Close-up of the active site of *Tc*PYK-F26BP-OX-Mg showing the metal coordination and stabilization of the small *α*-helix A*α*6′ by oxalate binding. The polypeptide chain is shown as a cartoon. Ligands and interacting residues are shown as sticks. Residue Phe213 which potentially favours the binding of oxalate in the same pose as the substrate PEP is also shown in stick format. Chain B of the protein is coloured yellow and chain D is blue. The divalent metal ion Mg^2+^ and monovalent ion K^+^ are shown as a green sphere and a purple sphere, respectively. The *Fo–Fc* electron densities for Mg^2+^ and K^+^ are shown as grey meshes contoured at 3.0 *σ* and 4.0 *σ*, respectively. Water molecules are shown as red spheres. Potential interatomic interactions are indicated by purple dashed lines and the relevant distances are given in ångströms. Detailed metal coordination is shown in the electronic supplementary material, figure S1.

### 4.2 Sequence identity explains different effector recognition

The *Tc*PYK amino acid sequence is 81% identical to *Tb*PYK and 76% to *Lm*PYK (electronic supplementary material, table S1), with residues involved in substrate and effector binding conserved (figure 2). This high degree of sequence conservation between trypanosomatids is reflected in their similar kinetic parameter values (table 1). *Tc*PYK and the F16BP-regulated human M2PYK are only 47% identical (electronic supplementary material, table S1), although the residues involved in substrate binding exhibit 100% sequence conservation (figure 2). The side chains involved in effector binding are much more variable and show only 57% sequence identity.

### 4.3 T- to R-state transition: a common pivot region

**Figure 4.**
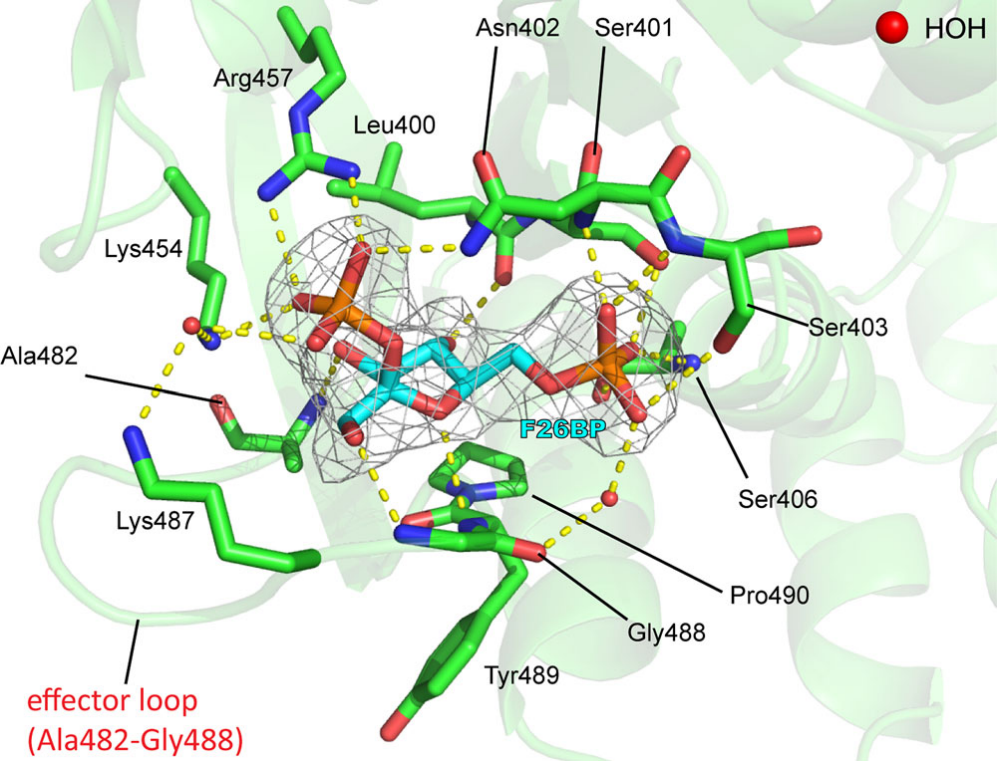
Close-up of the effector site of *Tc*PYK (*Tc*PYK-F26BP-OX-Mg, chain A) showing the binding mode of the activator F26BP. The protein is shown as a cartoon, except the residues involved in F26BP binding which are shown as sticks. The F26BP molecule is shown as sticks with an unbiased*Fo–Fc* election density map contoured at 4.0 *σ* (grey). Water molecules are shown as red spheres. Possible interactions involved in F26BP binding are indicated by dashed lines in yellow. The ordered effector loop (Ala482-Gly488) is also indicated. A schematic drawing showing the interatomic distances of the interactions is shown in the electronic supplementary material, figure S2.

**Figure 5.**
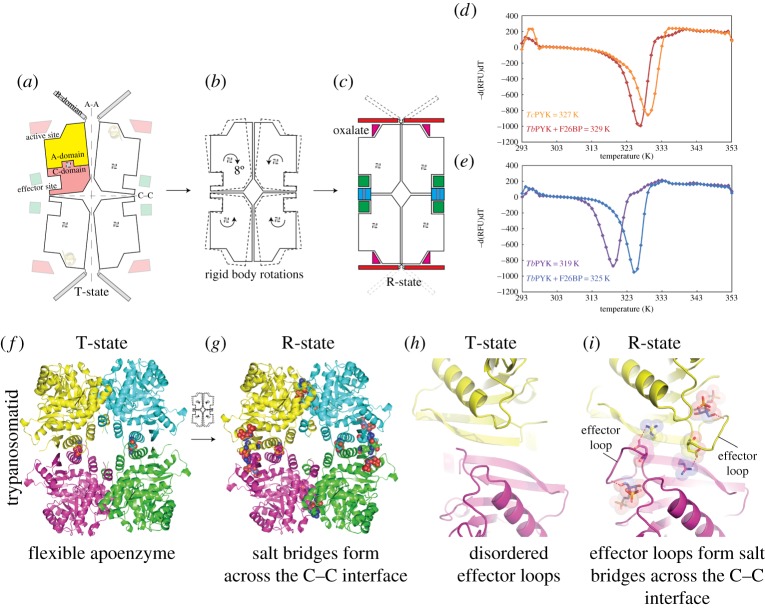
The T- to R-state allosteric transition results in the formation of inter-chain interactions and increased thermal stability. Schematic of the (*a*) T- and (*c*) R-state *Tc*PYK crystal structures. (*b*) Superposition of the T-state AC core structure onto the R-state AC core structure (all B-domains have been removed). The 8° rigid body rotations of the AC cores occurring during the T- to R-state transition have been indicated using arrows to show the direction of movement. (*d*) Thermal unfolding of *Tc*PYK and (*e*) *Tb*PYK in the absence and the presence of the effector molecule F26BP. (*f*) Cartoon representations of the T- and (*g*) R-state *Tc*PYK structures; (*h*,*i*) after rotation by 90°. Amino acids involved in inter-chain interactions are shown as spheres. The effector loops are disordered in the T-state but become ordered upon effector binding resulting in formation of salt bridges across the C–C interface.

Both T- and R-state *Tc*PYK X-ray crystal structures were obtained under near identical (pH 7.2) conditions (see table 2 for data collection and refinement statistics). In the R-state structure (*Tc*PYK-F26BP-OX-Mg), the active and effector sites are occupied by Mg^2+^-oxalate and F26BP, respectively (figures 3 and 4). In the T-state structure of *Tc*PYK (apo-*Tc*PYK), the effector loop (Ala482-Gly488) is disordered in the absence of F26BP binding. A superposition of the T- and R-state tetramers shows that the T- to R-state transition involves a rigid body rotation of subunits (excluding B-domains; AC cores; figure 5*a*–*c*), whereby the subunits pivot 8° (electronic supplementary material, table S2) around a region (residues 430–433) located at the base of the *αβ* barrel of domain A (figure 1*b*). In a similar way, *Lm*PYK experiences a rigid body rotation of its AC cores during the transition between T- and R-states [10]. A superposition of the *Tc*PYK and *Lm*PYK AC cores gave a C^*α*^ RMS fit of 0.7 and 0.6 Å for the T- and R-states, respectively.

**Table 2.** Data collection parameters, refinement and Ramachandran plot statistics. Values in parentheses are for the highest resolution shell.

	Apo-*Tc*PYK	*Tc*PYK-F26BP-OX-Mg
data collection and processing		
space group	*C*2	*I*422
unit-cell parameters (Å,°)	*a*=113.78, *b*=121.42, *c*=97.16	*a*=173.77, *b*=173.77, *c*=211.86
	*α*=*γ*=90.00, *β*=115.73	*α*=*β*=*γ*=90.00
solvent content (%)	53	65
wavelength (Å)	0.98	0.98
resolution (Å)	43.77−2.50	55.90−2.80
no. of reflections	252 349 (31 324)	553 645 (58 315)
no. of unique reflections	38 483 (5416)	39 095 (5770)
Wilson B-factor (Å^2^)	37.4	69.7
*R*_*merge*_ (%)	9.0 (25.7)	12.1 (51.5)
〈*I*/*σI*〉	13.7 (5.8)	20.1 (3.7)
*R*_*meas*_ (%)		
within *I*+/*I*−	10.5 (30.6)	12.6 (54.3)
all *I*+ and *I*−	10.4 (30.2)	12.6 (54.3)
*R*_*p*.*i*.*m*._(%)		
within *I*+/*I*−	5.5 (16.4)	3.3 (17.0)
all *I*+ and *I*−	3.9 (11.9)	3.3 (17.0)
completeness (%)	93.7 (90.5)	100.0 (100.0)
multiplicity	6.6 (5.8)	13.8 (10.1)
refinement statistics		
monomers in asymmetric unit	2	2
no. of reflections	36 536	38 066
*R*_*work*_/*R*_*free*_	16.06/22.28	17.39/21.63
no. of non-H atoms		
protein	7610	7417
ligands	12	52
metal ions	2	4
water	451	254
average B-factors (Å^2^)		
overall	30.39	54.01
protein	30.53	54.59
protein (excluding B-domains)	26.45	46.75
ligands	44.58	42.19
metal ions	46.96	46.84
water	27.86	39.60
RMS deviations		
bond lengths (Å)	0.01	0.01
bond angles (°)	1.02	1.03
Ramachandran plot		
favoured (%)	95.9	94.7
allowed (%)	99.9	99.7
no. of outliers	1^*a*^	3^*b*^

^*a*^The outlier residue is Thr296 in chain B, a key active site residue that is commonly found in this configuration in PYK structures.

^*b*^The outlier residues are Ile134 in chain A which is modelled in the poor electron density, and Thr296 in both chains.

By contrast, superposition of the AC core of active R-state *Tc*PYK with the equivalent human M2PYK structure (PDB ID: 3bjf) gave an average RMS fit of 2.3 Å. Besides the effector site, the major difference between these structures is an extended loop (inserted loop) between the C-terminal portion of helix A*α*2 and the N-terminal portion of strand A*β*3 (figure 1*a*). This extended loop which is also conserved in human M1PYK corresponds to six additional amino acid residues (Ser^100^-Asp-Pro-Ile-Leu-Tyr^105^) (figure 2) and seems to play a central role in regulating M2PYK activity. Mutation of Ile103 of human M2PYK to a tyrosine causes the formation of a monomeric form of M2PYK known as cytosolic thyroid hormone-binding protein p58 [34]. In addition, phosphorylation of Tyr105 has been shown to inhibit M2PYK activity [35]. Moreover, this region also comprises part of a known amino acid binding site in M1PYK (figure 1*a*). The pivot point is located close to the amino acid binding site and suggests that ligands binding to this site may affect the rocking motion of the AC cores and as a result modulate enzyme activity.

### 4.4 Effector loop conformation is responsible for allosteric control

**Figure 6.**
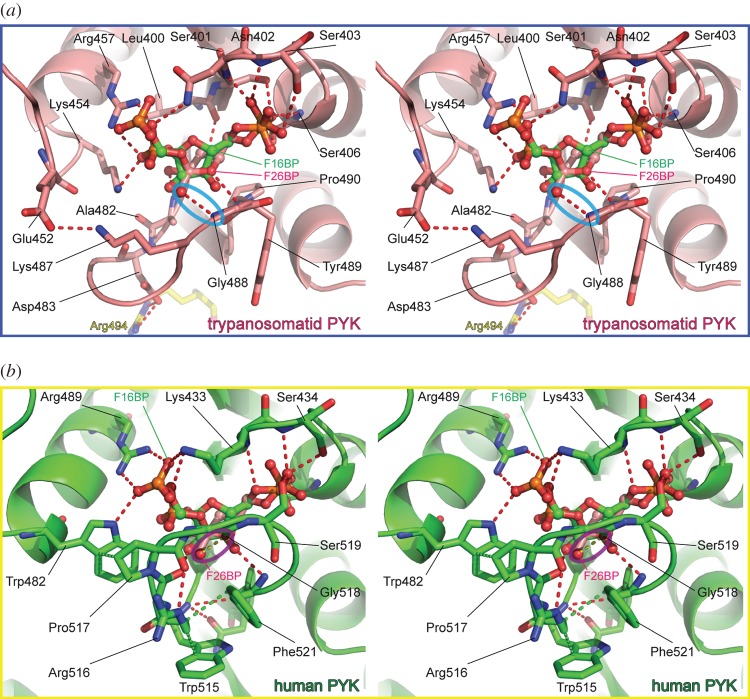
Stereo pictures of the F26BP and F16BP effector binding sites of trypanosomatid and human PYK. (*a*) A close-up view of the effector site of the *Tc*PYK-F26BP-OX-Mg structure showing the binding mode of F26BP (pink carbon atoms). The polypeptide chain is shown in cartoon format, with residues involved in F26BP binding shown as sticks. For comparison, the position of F16BP (green carbon atoms) from the human M2PYK-F16BP-OX-Mg structure (PDB ID: 3bjf) [36] is also shown. The two structures were superimposed by fitting corresponding C*α* atoms from the effector sites of both PYK structures. Effector binding stabilizes the effector loop (Ala482-Gly488) and the interaction of the 1′-CH_2_OH group (blue circle) of F26BP additionally holds the effector loop in a conformation that favours the formation of the allosteric salt bridge (Arg494…Asp483). (*b*) A close-up view of the effector site of the M2PYK-F16BP-OX-Mg structure showing the binding mode of F16BP. In contrast to the *Tc*PYK-F26BP-OX-Mg structure, the effector loop in M2PYK-F16BP-OX-Mg moves further from the C–C interface, essentially wrapping around the effector molecule. For comparison, the position of F26BP from the *Tc*PYK-F26BP-OX-Mg structure is also shown; the two structures were superimposed using selected corresponding C*α* atoms from the effector site. Interestingly, the 1′-CH_2_OH group belonging to F26BP clashes (less than 2 Å; purple circle) with the human effector loop position. Green dashed lines highlight stacking interactions.

The hydrogen bond interactions between F26BP and trypanosomatid PYKs are remarkably similar to those found in the F16BP–M2PYK complex (figure 6) which explains why *Tb*PYK, *Tc*PYK and *Lm*PYK are able to bind both effectors (table 1). However, higher concentrations (at least 1000-fold) of F16BP over the concentration of F26BP are required to induce a (weaker) allosteric response compared with F26BP. In the *Tc*PYK-F26BP-OX-Mg structure, the 2′- and 6′-phospho groups of the F26BP molecule form a series of hydrogen bonds similar to those of F16BP in the human M2PYK-F16BP-OX-Mg structure (cf. figure 6*a*,*b*), although the furanose ring adopts a significantly different pose. Pro490 in *Trypanosoma* PYKs (replaced by a threonine in the M2PYK-OX-F16BP structure) stacks with the furanose ring of F26BP positioning the 3′-OH and 4′-OH groups for hydrogen bond formation via the backbone nitrogen of Ala482 and the carbonyl of Leu390, respectively. The 1′-CH_2_OH group of F26BP forms a hydrogen bond via the backbone nitrogen of Gly488 (figure 6*a*), stabilizing the effector loop conformation.

By contrast, the effector loop of the M2PYK-F16BP-OX-Mg structure is stabilized by hydrogen bonds from 3′-OH and 4′-OH groups with the carbonyls of Arg516 and Gly518 and the backbone nitrogen of Phe521 and Gly514 (figure 6*b*). The conformation of the effector loop is further stabilized through a series of stacking interactions (Trp482, Pro517, Arg516, Trp515 and Phe521). These subtly different effector interactions in the two structures result in different conformations of effector loop: in the *Tc*PYK structure the 1′-CH_2_OH group prevents the effector loop from folding up around F26BP and stabilizes the loop in a position that favours the formation of an inter-chain salt bridge (Asp483…Arg494) across the C–C interface (figure 5*g*,*i*), an interaction essential for allosteric control [10]. Instead of forming additional salt bridges, the binding of F16BP to human M2PYK stabilizes a lysine-mediated ‘peg-in-hole’ connection across the C–C interface [16].

### 4.5 Increased thermal stability correlates with increased *k*_cat_/*S*_0.5_

**Figure 7.**
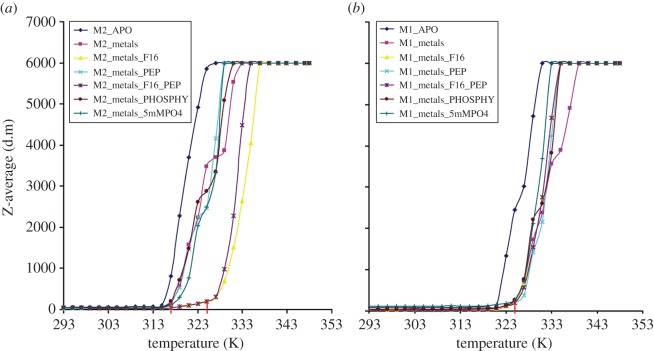
Stabilization of the human M2PYK tetramer by binding of F16BP. (*a*) Thermal shift assay results for human M2PYK and (*b*) human M1PYK complexes monitored by dynamic light scattering. Assays were performed in 20 mM TEA (pH 7.2) and 10% glycerol. Samples were as follows: M2_APO, M2PYK in buffer only; M2_metals, M2PYK in buffer containing 50 mM MgCl_2_ and 100 mM KCl; M2_metals_F16, M2PYK in buffer containing metals and 1 mM F16BP; M2_metals_PEP, M2PYK in buffer containing 50 mM MgCl_2_, 100 mM KCl and 1 mM PEP; M2_metals_F16_PEP, M2PYK in buffer containing 50 mM MgCl_2_, 100 mM KCl, 1 mM PEP and 1 mM F16BP; M2_metals_PHOSPHY, M2PYK in buffer containing 50 mM MgCl_2_, 100 mM KCl and 1 mM phosphotyrosine; M2_metals_5mMPO4, M2PYK in buffer containing 50 mM MgCl_2_, 100 mM KCl and 5 mM phosphate. Corresponding samples are shown for M1PYK in (*b*). The *T*_m_ for M2PYK is 315 K in the absence of F16BP, shifting by 10 K to 325 K in the presence, similar to that of (unligated) M1PYK (325 K).

The stabilizing effects of PYK cofactors, inhibitors and metals were analysed using thermal shift assays. An increase in melting temperature (*T*_m_) reflects an increase in structural order and reduced conformational flexibility. Addition of the effector molecule (F26BP) to apo-*Tc*PYK or apo-*Tb*PYK increased the melting temperature by 2 K and 8 K, respectively (figure 5*d*,*e*). The addition of F16BP to M2PYK also showed a dramatic increase in the *T*_m_ from 315 to 325 K (figure 7*a*), close to the *T*_m_ of the non-allosterically regulated M1PYK, for which all complexes had identical *T*_m_ values of 325 K (figure 7*b*). This increase in *T*_m_ upon effector binding correlates with a 10- to 16-fold increase in specificity constant (*k*_cat_/*S*_0.5_) (table 1).

### 4.6 Changes in the C–C interface account for increased enzyme stability and *k*_cat_/*S*_0.5_

Previous site-directed mutational studies on *Lm*PYK have shown that the increased stability is the result of a series of stabilizing salt bridges formed across the C–C tetramer interface [10]. These interactions are also observed in the *Tc*PYK-F26BP-OX-Mg structure (figure 5*i*), indicating a similar mechanism of regulation. Upon addition of effector an increase in *T*_m_ is observed for all trypanosomatid PYKs correlating with a 10- to 14-fold increase in *k*_cat_/*S*_0.5_, clearly demonstrating the relationship between rigidity and activity.

**Figure 8.**
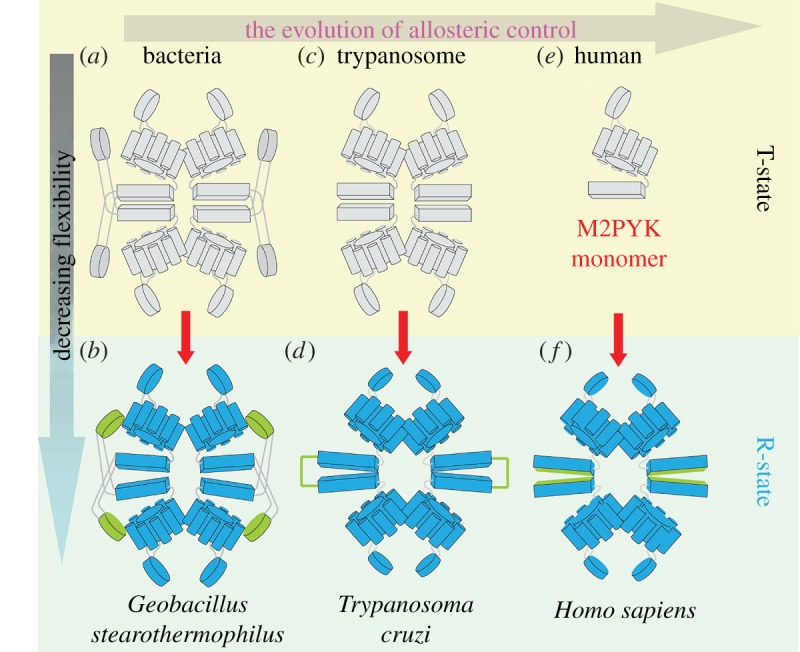
The evolution of allosteric control of PYK. Schematic of the effector induced T- to R-state allosteric transition. This transition results in the formation of molecular bridges between chains creating a more rigid, highly active R-state conformer. (*a*) The ‘inactive’ *Geobacillus stearothermophilus* PYK (*Gs*PYK) T-state tetramer (PDB ID: 2E28). An extra domain (residues 474–587) corresponding to the effector loop in the trypanosomatid structures is shown in green. This domain was predicted to have a hinge type movement (HingeProt) [38] and its movement probably mimics the trypanosomatid effector loop, forming molecular bridges between chains (as shown in *b*) and the thermally stable highly active R-state tetramer observed in solution. (*c*) In trypanosomatid PYKs F26BP binding stabilizes the effector loop, resulting in the formation of a series of stabilizing salt bridge interactions across the C–C interface (as shown in *d*), generating the highly stable (*ΔT*_*m*_=2–8 K) R-state tetramer. (*e*) The ‘inactive’ form of the human M2PYK is monomeric (*T*_m_=315 K) in solution shifting to tetrameric (*T*_m_=325 K) upon F16BP binding. Addition of F16BP reduces thermal vibration along the C–C interface and locks the effector loops, stabilizing the highly active tetramer conformation.

The addition of effector molecule to apo-M2PYK dramatically increased the *T*_m_ from 315 to 325 K (figure 7*a*) with a concomitant 16-fold increase in *k*_cat_/*S*_0.5_ (table 1). A recent analysis of the oligomeric state of M2PYK provided a novel mechanism for tetrameric stabilization, whereby M2PYK exists in a monomer–tetramer equilibrium [16,37], and binding of the allosteric effector F16BP stabilizes the peg-in-hole connection across the C–C interface of the tetramer to shift this equilibrium to a predominantly tetrameric state (figure 8*e*,*f*). Addition of F16BP reduces thermal vibration along the C–C interface (apo-M2PYK *T*_m_=315 K, shifting to *T*_m_=325 K for effector bound) and locks the effector loops, stabilizing the active tetramer conformation. We suggest that the 16-fold increase in specificity constant results from stabilization of the tetramer and promotes conformational states which favour substrate binding (8.6-fold decrease in S_0.5_) and/or catalytic activity and/or product release. In addition, restriction in its conformational states reduces the ensemble of inactive conformational states thereby favouring substrate binding. The activity of M1PYK also correlates with tetrameric stability as the X-ray structure (PDB: 3SRF) shows a tighter peg-in-hole binding across the C–C interface compared with M2PYK which locks the enzyme in a constitutively active tetramer conformation (figure 7*b*).

Tetrameric stabilization by means of effector binding has also been observed for the bacterial PYK from *G. stearothermophilis* (*Gs*PYK). *Gs*PYK exists as a tetramer and is regulated by the allosteric activator ribose 5-phosphate (R5P) [8]. Lovell *et al.* have shown that the addition of the effector molecule results in a highly thermostable *Gs*PYK conformation, demonstrating the increased rigidity of effector bound complexes. Determination of the crystal structure of *Gs*PYK [2] revealed that the C-terminal extension forms an extra domain (domain C') in place of the trypanosomatid PYK effector loop (figure 8*a*). This domain is predicted to have a hinge-like movement, possibly interacting with adjacent monomers and forming stabilizing bridges across the C–C interface (figure 8*b*), similar to those observed for the salt bridge effector loops of trypanosomatid PYKs (figure 8*d*).

### 4.7 Evolution of allosteric control mechanisms

Trypanosomatid PYKs are allosterically regulated by the binding of F26BP, human M2PYK (and many other PYKs) by F16BP, and certain bacterial PYKs by AMP or monophosphorylated sugars. While the regulatory molecules and molecular mechanisms may vary between species the underlying principle of tetrameric stabilization in response to effector binding is conserved.

Three types of allosteric mechanisms are found in evolutionarily divergent species of PYK (figure 8). (i) Bacterial PYKs as exemplified by the *Gs*PYK tetramer (figure 8*a*,*b*) are stabilized by the rotation of a series of additional domains (domain C') which bridge across the C–C interface thereby stabilizing the tetramer. (ii) Trypanosomatid PYKs (figure 8*c*,*d*) possess simple loops (effector loops) which again form a series of bridges across the C–C interface enhancing tetramer stability. (iii) Human M2PYK (figure 8*e*,*f*) has evolved in a different direction whereby it is able to dissociate into inactive monomers, and the active tetramer is formed and stabilized in response to effector binding. The allosteric characteristics observed for all PYKs suggest that effector bound PYKs have higher thermal stability and greater enzymic activity. Selective disruption of these allosteric mechanisms may provide new and specific drug targets which avoid the problem of developing selective inhibitors against similar active sites. Such inhibitors/activators could be used to tackle trypanosomiasis, cancer (M2PYK [39]), anaemia (RPYK [40]) and bacterial infection (methicillin-resistant *Staphylococcus aureus* PYK [6]).

## Supplementary Material

PYK_allosteric_strategies_supplementary Additional figures and sequence analysis

## Data Accessibility

Protein crystal structures deposited with the PDB: *Tc*PYK apoenzyme, 4krz; *Tc*PYK-F26BP-OX-Mg, 4ks0.
