# Supplementary material for: Structures of pyruvate kinases display evolutionarily divergent allosteric strategies
Source: R Soc Open Sci. 2014 Sep 24;1(1):140120. doi: 10.1098/rsos.140120 (PMC4448766; doi:10.1098/rsos.140120)
Supplement: PYK_allosteric_strategies_supplementary Additional figures and sequence analysis [file rsos140120supp1.docx]

**Structures of pyruvate kinases display evolutionarily divergent allosteric strategies**

**Supplementary Data**

**Table S1**

Pairwise protein sequence comparisons of trypanosomatid, human M2, human M1, yeast, *E. coli* and *G. stearothermophilus* PYKs

|  |  | *Tc*PYK | *Tb*PYK | *Lm*PYK | *Hs*M2PYK | *Hs*M1PYK | *Sc*PYK | *Ec*PYK | *Gs*PYK |
| --- | --- | --- | --- | --- | --- | --- | --- | --- | --- |
| *Tc*PYK |  | 100 | 81 | 76 | 47 | 47 | 48 | 42 | 35 |
| *Tb*PYK |  |  | 100 | 74 | 48 | 47 | 48 | 42 | 35 |
| *Lm*PYK |  |  |  | 100 | 47 | 48 | 48 | 42 | 34 |
| *Hs*M2PYK |  |  |  |  | 100 | 96 | 49 | 44 | 36 |
| *Hs*M1PYK |  |  |  |  |  | 100 | 48 | 44 | 36 |
| *Sc*PYK |  |  |  |  |  |  | 100 | 43 | 34 |
| *Ec*PYK |  |  |  |  |  |  |  | 100 | 42 |
| *Gs*PYK |  |  |  |  |  |  |  |  | 100 |

The pairwise sequence analysis was obtained by using the EMBL-EBI web server (EMBOSS Stretcher: <http://www.ebi.ac.uk/Tools/psa/emboss_stretcher/>). Values are overall percentage sequence identities.

*Tc, Trypanosoma cruzi; Tb, Trypanosoma brucei; Lm, Leishmania mexicana; Hs, Homo sapiens; Sc, Saccharomyces cerevisiae* (baker’s yeast)*; Ec, Escherichia coli; Gs, Geobacillus stearothermophilus*

**Table S2**

The AC-core rigid-body rotation from T- to R-state of *Tc*PYK

| T-state | R-state |  | Average  RMS (Å) † | Rotation  Angle (^o^)‡ |
| --- | --- | --- | --- | --- |
| Apo-*Tc*PYK | *Tc*PYK-F26BP-OX-Mg |  | 2.39 | 8.2±0.3 |

†Average C^α^ RMS differences between T- and R-state tetramer structures (AC cores). All four AC cores (A- and C-domains) from the apo-*Tc*PYK tetramer were simultaneously superposed onto all four AC cores from *Tc*PYK/F26BP/OX/Mg tetramer.

‡Calculated rotation angle with standard deviation.


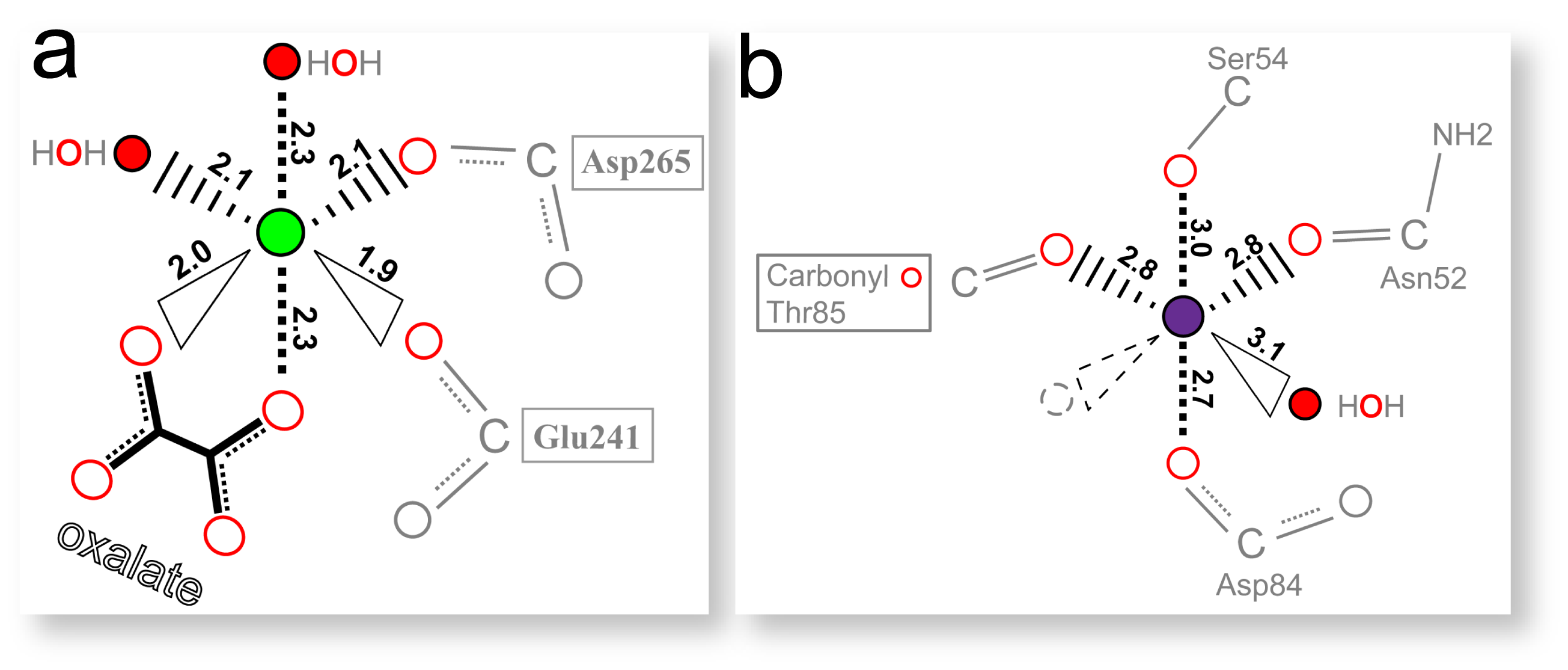


**Figure S1**

Schematic representations are shown for Mg^2+^ coordination (a) and K^+^ coordination (b) in the active site. The octahedral geometry for K^+^ is not completed and the missing coordination is represented by dashed lines. The interatomic distances for the interactions are given in Ångstroms.


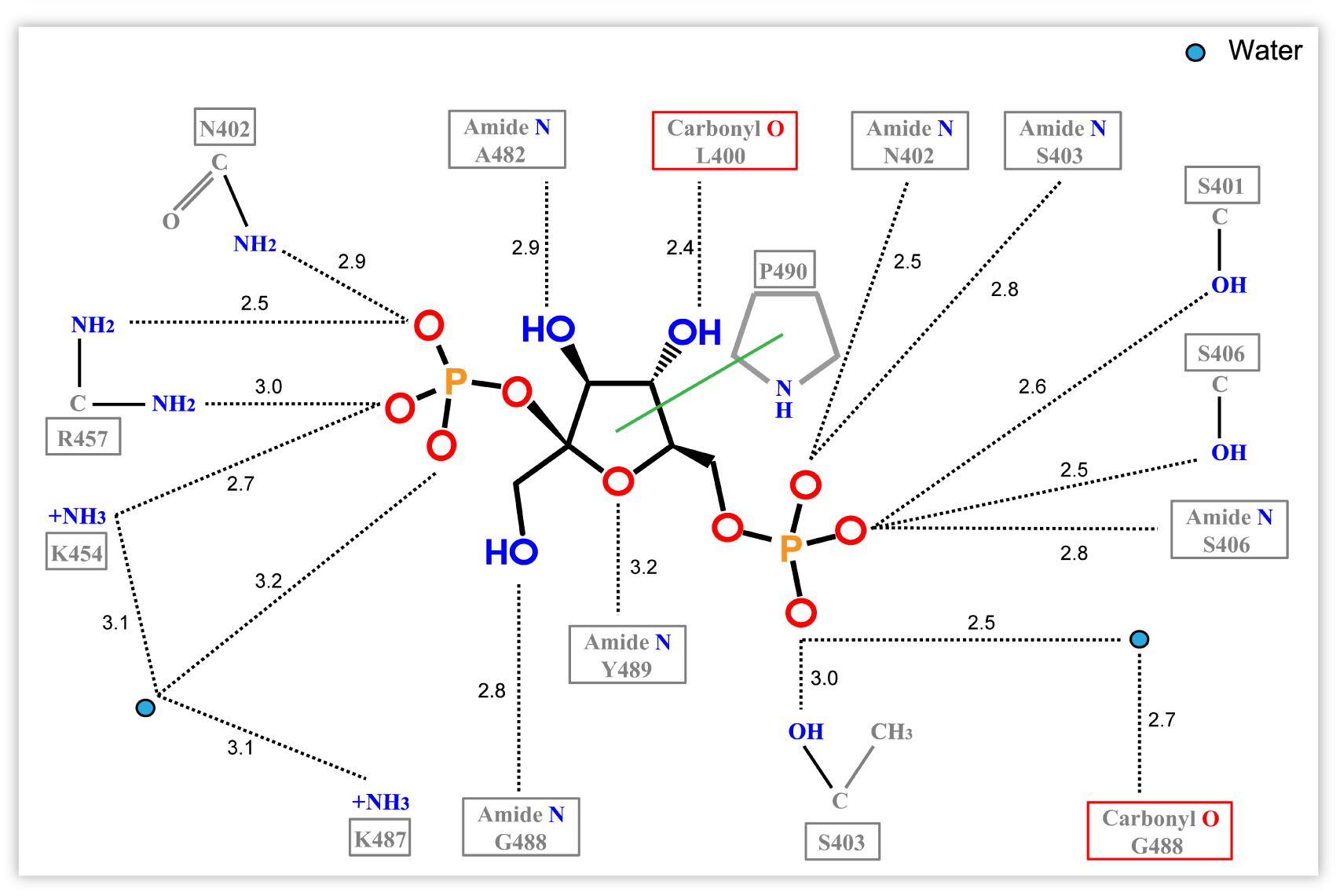


**Figure S2**

A schematic drawing showing the estimated interactions at the *Tc*PYK effector site. The interatomic distances for the interactions are given in Ångstroms.
